# p90RSK2, a new MLCK mediates contractility in myosin light chain kinase null smooth muscle

**DOI:** 10.3389/fphys.2023.1228488

**Published:** 2023-09-13

**Authors:** Jaspreet Kalra, Mykhaylo Artamonov, Hua Wang, Aaron Franke, Zaneta Markowska, Li Jin, Zygmunt S. Derewenda, Ramon J. Ayon, Avril Somlyo

**Affiliations:** ^1^ Department of Molecular Physiology and Biological Physics, University of Virginia, Charlottesville, VA, United States; ^2^ Vaccine Research Center, National Institute of Allergy and Infectious Diseases, National Institutes of Health, Bethesda, MD, United States; ^3^ Sentara Martha Jefferson Hospital, Charlottesville, VA, United States; ^4^ Brain Surgery Worldwide, Atlanta, GA, United States; ^5^ Department of Orthopedics, University of Virginia, Charlottesville, VA, United States

**Keywords:** smooth muscle, MLCK, *mylk1*, p90 ribosomal S6 kinase, RSK2, PDK1, Pyk2

## Abstract

**Introduction:** Phosphorylation of smooth muscle (SM) myosin regulatory light chain (RLC_20_) is a critical switch leading to SM contraction. The canonical view held that only the short isoform of myosin light chain kinase (MLCK1) catalyzed this reaction. It is now accepted that auxiliary kinases may contribute to vascular SM tone and contractility. We have previously reported that p90 ribosomal S6 kinase (RSK2) functions as such a kinase, in parallel with MLCK1, contributing ∼25% of the maximal myogenic force in resistance arteries. Thus, RSK2 may be instrumental in the regulation of basal vascular tone and blood pressure. Here, we take advantage of a MLCK1 null mouse (*mylk1* ^−/−^) to further test our hypothesis that RSK2 can function as an MLCK, playing a significant physiological role in SM contractility.

**Methods:** Using fetal (E14.5-18.5) SM tissues, as embryos die at birth, we investigated the necessity of MLCK for contractility and fetal development and determined the ability of RSK2 kinase to compensate for the lack of MLCK and characterized its signaling pathway in SM.

**Results and Discussion:** Agonists induced contraction and RLC_20_ phosphorylation in *mylk1* ^−/−^ SM was attenuated by RSK2 inhibition. The pCa-tension relationships in permeabilized strips of bladder showed no difference in Ca^2+^ sensitivity in WT vs *mylk1* ^−/−^ muscles, although the magnitude of force responses was considerably smaller in the absence of MLCK. The magnitude of contractile responses was similar upon addition of GTPγS to activate the RhoA/ROCK pathway or calyculinA to inhibit the myosin phosphatase. The Ca^2+^-dependent tyrosine kinase, Pyk2, contributed to RSK2-mediated contractility and RLC_20_ phosphorylation. Proximity-ligation and immunoprecipitation assays demonstrated an association of RSK2, PDK1 and ERK1/2 with MLCK and actin. RSK2, PDK1, ERK1/2 and MLCK formed a signaling complex on the actin filament, positioning them for interaction with adjacent myosin heads. The Ca^2+^-dependent component reflected the agonist mediated increases in Ca^2+^, which activated the Pyk2/PDK1/RSK2 signaling cascade. The Ca^2+^−independent component was through activation of Erk1/2/PDK1/RSK2 leading to direct phosphorylation of RLC_20_, to increase contraction. Overall, RSK2 signaling constitutes a new third signaling pathway, in addition to the established Ca^2+^/CaM/MLCK and RhoA/ROCK pathways to regulate SM contractility.

## Introduction

In vascular smooth muscle (VSM), contraction is initiated by phosphorylation of the regulatory light chain (RLC_20_) of SM myosin II on Ser19 by the SM myosin light chain kinase (smMLCK, referred to hereafter as MLCK). MLCK is activated by Ca^2+^-bound calmodulin (CaM). (Gallagher et al., 1992; Hartshorne et al., 1998). In this way, Ca^2+^ influx promotes phosphorylation of RLC_20_, activating SM myosin II and its binding to actin filaments, thereby initiating cyclic ATP-dependent cross-bridge cycling. The activity of MLCK is counterbalanced by that of the RLC_20_ phosphatase (MLCP) which induces relaxation. MLCP itself is regulated by phosphorylation, specifically by the RhoA-dependent Ser/Thr kinase ROCK. The latter is activated by the small cytosolic GTPase RhoA, downstream of G-protein coupled receptors. This pathway sustains contraction as intracellular [Ca^2+^] decreases, a phenomenon known as Ca^2+^-sensitization (Somlyo and Somlyo, 2003).

The widely expressed *mylk1* gene contains alternative promotors giving rise to three proteins: the long (∼220 kDa) non-muscle myosin II light chain kinase, expressed in various cell types; the short (∼130 kDa) isoform unique to SM acting on the myosin II isoform; and the non-catalytic kinase related protein also known as telokin (17 kDa) (Kamm and Stull, 2001; Herring et al., 2006). Telokin is a small protein whose sequence is identical to the COOH terminus of SM ∼220 and ∼130 kDa MLCKs and is independently expressed in SMs through a promotor located within an intron of the *MYLK1* gene. Long form/short form/telokin will be used interchangeably with 220/130/17 kDa isoforms throughout the manuscript. In the classical view of SM contraction, MLCK was the sole protein kinase regulating RLC_20._ Over the past few decades, strong evidence emerged that this scheme is oversimplified. For example, direct evaluation of MLCK activity in bladder SM from a CaM-sensor-MLCK transgenic mouse model demonstrated that only about 50% of carbachol-induced force and RLC_20_ phosphorylation was accounted for by MLCK activity (Isotani et al., 2004). Thus, additional mechanisms exist for increasing both force and RLC_20_ phosphorylation. The possibility of other auxiliary kinases regulating SM contractility is of enormous significance, because reactivity and tone of vascular SM affects vascular resistance, and consequently blood pressure (BP) (Bohr and Sitrin, 1970; Goulopoulou and Webb, 2014; Touyz et al., 2018). Studies in mice with tamoxifen-induced, SM-specific partial MLCK knockout have shown that BP is significantly reduced and salt-induced hypertension is abolished (He et al., 2008; He et al., 2011). However, therapeutic inhibition of MLCK is not a practical option, in part because the ubiquitously expressed non-muscle myosin shares the same catalytic domain and because of its major role in contractile activity of all SM-containing hollow organs. As a result, there is ongoing research to identify auxiliary, regulatory protein kinases in VSM as potential targets for antihypertensive therapy, as even small reductions of BP can have clinically significant effects.

Several potential kinases have been identified that target RLC_20_, including ROCK (Amano et al., 1996), the ROCK-related citron kinase (Yamashiro et al., 2003), the zipper interacting protein kinase (ZIPK) (Murata-Hori et al., 1999; Niiro and Ikebe, 2001), integrin-linked kinase (ILK) (Deng et al., 2001; Walsh, 2011), the Inhibitor κΒ kinase 2 (IKK2) (Ying et al., 2013) and the PIM3 (Proviral Integration site for Moloney murine leukemia virus) kinase (Carlson et al., 2018). These kinases have been shown to phosphorylate both Ser19 and the adjacent Thr18 *in vitro*. However, the specific physiological implications of *in vitro* studies are not fully understood. There is evidence that PIM/ZIPK contribute to contraction and maintenance of vascular tone, and that inhibitors of these kinases can reduce BP in mouse models (Carlson et al., 2018). Further, IKK2 deficient mice have been found to have decreased aortic contractile responses and reduced responses to hypertensive vasoconstrictors, suggesting a potential role in regulating vascular function and BP (Ying et al., 2013).

The p90 ribosomal S6 kinase isoform 2 (RSK2), encoded by the gene *RPS6KA3*, was another kinase observed to phosphorylate MLCK *in vitro* on Ser19 (Suizu et al., 2000). We recently demonstrated that RSK2 contributes to basal vascular tone, myogenic vasoconstriction of resistance arteries and blood pressure (BP) (Artamonov et al., 2013a; Artamonov et al., 2018). Our research revealed that this is mediated by direct phosphorylation of RLC_20_ as well as phosphorylation of the Na^+^/H^+^ exchanger, NHE1, which leads to an alkalinization of the SM cytosol and increased cytosolic [Ca^2+^], activating MLCK and/or other Ca^2+^-dependent kinase(s) (Artamonov et al., 2018). RSK kinases (four isoforms) are atypical in that they have two catalytic domains: the functionally important N-terminal catalytic domain (NTKD) and the regulatory C-terminal (CTKD). The activation of RSK2 in cells is triggered by the phosphorylation of CTKD by ERK1/2 kinases on several residues, including Ser386, which creates a docking site for 3-phosphoinositide-dependent protein kinase 1 (PDK1). The latter phosphorylates and activates NTKD on Ser227 (Anjum and Blenis, 2008; Pearce et al., 2010; Lara et al., 2013). We observed that increased intraluminal pressure in resistance vessels to induce myogenic vasoconstriction results in phosphorylation of Ser227 of endogenous RSK2; moreover, vasoconstriction and Ser227 phosphorylation as well as RLC_20_ phosphorylation are blocked by RSK inhibitors BI-D1870 and LJH685 (Artamonov et al., 2013a; Artamonov et al., 2018). RSK2 signaling accounts for approximately 25% of the maximal myogenic constriction in normal mouse resistance arteries, implicating RSK2 as a significant player in vasoconstriction under physiological conditions. Accordingly, BP was significantly lower and the myogenic response suppressed in mice with global RSK2 knockout (Artamonov et al., 2018). Data suggest, RSK2 may account for the contractility observed in the absence of MLCK and could be a viable drug target for hypertension.

To understand the role of RSK2 in SM function in a rigorous manner, it was necessary to conduct experiments utilizing a mouse model with complete *mylk1* knockout. Previously, we reported generation of a global *mylk1* knockout in mice, by disrupting promotor sequences located within intron 27 (Somlyo et al., 2004). Although the embryos developed to full size, they died within 1–5 h after birth. This KO mouse will subsequently be referred to as *mylk1* ^−/−^. The expression of all three *mylk1* products in these mice was abolished, as evidenced by Western blot and polymerase chain reaction analysis. Unfortunately, at the time, we did not pursue further experimental characterization of the SM tissues from these embryos; we completed the work now and are reporting the full results. Further, we gained insights into the mechanisms responsible for contractions and RLC_20_ phosphorylation that occur in the absence of MLCK, including importantly the role of RSK2. Our data corroborate that RSK2 mediates these contractions. Additionally, we present compelling evidence that the apparent Ca^2+^-dependence of RSK2 is mediated by the upstream activity of the Ca^2+^ activated tyrosine kinase, Pyk2, which leads to the activatory phosphorylation of PDK1 and/or ERK1/2, with consequent upregulation of RSK2. This is consistent with previous findings about the physiological role of Pyk2 in SM. (Eguchi et al., 1999; Taniyama et al., 2003). We also report that similar to MLCK, RSK2 binds to actin and to MLCK forming a signaling hub with PDK1 and ERK1/2 on the actin filament, ideally positioned to interact with the myosin RLC_20_.

Our findings provide further support of an important role for RSK2 signaling in vascular SM contractility, and provide rationale for the observed contractility of SM in tissues with *mylk1* knockout (*mylk1* ^*−/−*^).

## Materials and methods

### *Mylk1* ^*−/−*^ mice

Briefly a targeted construct that includes a loxP flanked neomycin selection cassette was inserted in intron 27 of the *mylk1* gene, that is located between exons encoding the calmodulin binding domain in the kinase; the construct was designed to allow Cre recombinase disruption of the telokin promoter sequence that included a CArG element (Somlyo et al., 2004). The insertion of the full length targeted construct, that includes the loxP flanked neomycin selection cassette, caused disruption of the entire gene resulting in a complete loss of MLCK (both 130- and 220-kDa isoforms and the non-catalytic 17-kDa telokin) in homozygous animals. To make the most of the limited number of *mylk1* ^*−/−*^ embryos, and the very small size of individual tissue samples, we harvested abdominal aorta, umbilical artery, and bladder from each of the 8–12 embryos as we waited for the PCR result to identify which embryos were *mylk1* ^*−/−*^ or *mylk1* ^*+/+*^. Some aortas were used to generate cultured aortic SM cells, other tissues were used for phosphorylation and contractility studies. The tissues were very fragile and did not survive mounting for contractility studies. Both *mylk1* ^*−/−*^ and *mylk1* ^*+/+*^ muscles were run as pairs for comparisons. This necessitated using all the aorta, umbilical arteries, and bladders from all the *mylk1* ^*−/−*^ and *mylk1* ^*+/+*^ embryos on the same day in order to get a sufficient number of experiments for a given protocol. This accounts for different SM tissues being used for the different assays (Table 1). All animal studies were performed under protocols that comply with the Guide for the Care and Use of Laboratory Animals and were approved by the Animal Care and Use Committee at the University of Virginia.

**TABLE 1:** E18 *mylk1* samples and assays.

E18 samples	Assay
Bladder	Contractility
	• High K^+^
	• pCa tension
	• GTPγS
	• ACh/RSK inhibitors
Umbilical artery	Contractility
	• High K^+^
	• Calyculin A
Aorta	Cultured SMCs
	• RLC_20_ phosphorylation
	• ERK1/2, RSK2, PDK1, Pyk2 Immunoblotting
	• PLA assay
	• Immunoprecipitation
	• Collagen gel

### Genotyping

PCR analysis was performed on tails or limbs of weaned pups and embryos (E18.5). Primers for *mylk1 and RPS6KA3* are detailed in Table 2.

**TABLE 2:** Genotyping primers.

*Mylk1*	Forward (Telokin P1): 5′-AGA AGG AAA CTG AAG CCT GGA GAG GTC AAG-3′
	Reverse (Telokin P3): 5′-ACT GTC AGC GTG TCC GAA GAT GTT CGG AGA ATG G-3′
	Forward (NEO1): 5′-CTT GGG TGG AGA GGC TAT TC-3′
	Reverse: (NEO2): 5′-AGG TGA GAT GAC AGG AGA TC-3′
Rps6ka3	Forward (RSK2): 5′ TCT CTC CTG TAT TTC CTT TCA GG 3′
	Reverse (RSK2): 5′ CT GAC CAC CAG GAA ACC ACA 3′
	Forward (NEO170): 5′ TGA ATG AAC TGC AGG ACG AG 3′
	Reverse (NEO684): 5′ GC AAC CGA TGA GCA CTA TAA 3′

### *Mylk1* ^−/−^ and *RPS6KA3* ^−/−^ primary aortic cells

Cultured smooth muscle cells were prepared from abdominal aortas from *mylk1* ^−/−^ and WT E18.5 mice and 2-month-old WT, *RPS6KA3* ^*−/−*^ mice, described previously (Artamonov et al., 2018), cleaned of adventitia, cut into 1 mm^2^ pieces, and left undisturbed for 10 days in culture medium. The *mylk1* ^−/−^ cells were grown and cultured in Amniomax c100 (1x) medium (Gibco, Billings, Montana, United States, catalog no: 17001-074), supplemented with 10% AmniomaxTM (Gibco catalog no: 12556-023). *RPS6KA3* ^−/−^ aortic cells were cultured in DMEM (Gibco catalog no: 11965-092) supplemented with 10% heat inactivated FBS (Gibco catalog no: 10438026). Cells were maintained at 37°C and 5% CO_2_. The absence of RSK2 and MLCK proteins was confirmed by gel electrophoresis and Western blotting. Cells were identified as SM cells based on the presence of SM myosin heavy chain expression detected on western blots. *Mylk1* ^*−/−*^ and *RPS6KA3* ^*−/−*^ lacked expression of *Mylk or RPS6KA3* (Supplementary Figures S1A, B respectively).

### Histology

After fixation in buffered formalin, alcohol dehydration and paraffin embedding, 4–5 μm sections of paraffin-embedded tissues were processed and stained with Hematoxylin and Eosin and mounted with Dpx mounting media (Cytoseal XYL, catalog no: 18009, Ellectron Microscopy Sciences, Hatfield, PA, United States).

### RLC_20_ phosphorylation

*Mylk1* ^−/−^ and WT cells were serum starved for 24 h, then stimulated with serum for 5 min in presence of either lysophosphatidic acid, LPA (0.5 μM) for 2.5 min, or with U46619 (1 μM) for 30 s in the presence and absence of LJH685 (10 μM), added 15 min before serum. (LPA stock solution 2.29 mM in 3 mg/mL BSA, catalog no. L7260, Sigma-Aldrich, St. Louis Mo. United States; U46619 stock solution 5 mg in 500 μL methyl acetate, catalog no 16450, Cayman Chemicals, Ann Arbor, Mi, United States; LJH685 stock solution 10 mM in DMSO, catalog no. 19913, Cayman Chemicals) Protein was precipitated using 10% trichloroacetic acid (TCA)/acetone (Neppl et al., 2009) and solubilized in 2x Laemmli buffer and prepared for gel electrophoresis. *Mylk1* ^−/−^ and WT embryonic bladders (E14.5–18.5) were stimulated with LPA (0.5 μM) for 5 min, flash frozen and freeze-substituted in 10% TCA/acetone at −80°C (Kitazawa et al., 1991). Frozen strips were then washed in acetone and dried, homogenized in sample buffer in a glass-glass, hand-operated homogenizer, and phosphorylation was estimated by Western blotting. For imaging, the membranes were blocked with the Intercept^®^ Odyssey blocking buffer (LI-COR). The ratio of phospho-RLC_20_ to total RLC_20_ was determined by immunoblotting with primary antibodies. Primary antibodies were visualized using secondary antibodies conjugated to either Alexa Fluor 680 (Thermo Fisher) or IRDye^®^ 800 (LI-COR) for Odyssey imaging.

### Pyk2 and PDK1 phosphorylation

Aortic SM cells were serum starved for 24 h, then co-incubated in presence/absence of selective Pyk2 inhibitor PF4618433 (10 μM) for 15 min. Thereafter, cells were stimulated in the presence or absence of AngII (1 μM) for 1 min. Protein was precipitated using 10% TCA and acetone and solubilized in 200 μL of 2x Laemmli buffer with 4% SDS. Cell were scraped off the plates, and samples were boiled at 95°C for 10 min then agitated overnight at 4°C and prepared for gel electrophoresis or stored at −80°C.

### Smooth muscle force measurements

Helical strips of abdominal aorta, umbilical arteries, or bundles from bladder taken from E14.5–18.5 embryos were cut and mounted on a bubble chamber (Kitazawa et al., 1991) in HEPES Krebs solution at room temperature, for isometric force measurements in response to agonists or high [K^+^] (137.4 mM KCL substituted for 137.4 mM NaCl). pCa-force relationships were obtained following permeabilization with 17.5 μg/mL *Staphylococcus aureus* α-toxin (List Biologica Laboratories, Campbell, CA, United States) by increasing [Ca^2+^] from pCa 6.3 to pCa 5.5 with each force normalized to the maximal force developed by the same strip at pCa 4.5 (Khromov et al., 2006). Ca^2+^-sensitization through the RhoA/ROCK signaling pathway was assayed by addition of 10 μM GTPγ S to α-toxin (500 U/mL) permeabilized bladder SM strips in pCa 6.3 solution (Kitazawa et al., 1989). Once force had reached a plateau, 5 and 10 μM Y27632 was added to inhibit ROCK and relaxation was measured. pCa solutions and α-toxin permeabilization are described elsewhere (Kitazawa et al., 1991). RSK2 signaling was determined by cumulative additions of carbachol in the presence or absence of 1 μM BiD1870 or DMSO diluent. Ca^2+^-independent force development in *mylk1* ^−/−^ embryonic arteries and bladder was induced by the MLCP inhibitor calyculin A 100 nM in the absence of Ca^2+^ (pCa 9.0).

### Immunoprecipitation assays

Embryonic WT, *mylk1* ^−/−^ and *RPS6KA3* ^−/−^ cells were serum starved for 24 h and stimulated with or without serum for 5 min prior to centrifugation for 10 min at 4°C, 20,000 g. Cell lysates were prepared in 1% Triton X-100 TBS buffer. The supernatant was applied to Protein G Sepharose 4 Fast Flow beads (GE Healthcare) preincubated with or without MLCK mouse monoclonal antibody (Sigma Aldrich, St. Louis, MO, United States) and incubated at 4°C for 1 h. After centrifugation, beads were washed (four times) with Tris buffer containing 0.1% triton X100 and proteinase inhibitor cocktail, Thermo, Invitrogen and solubilized in 2x Laemmli buffer and boiled at 95°C for 5 min. Extracted proteins were separated by SDS-PAGE at 70 V using 1.5 mm thick, 8% polyacryamide gel for Western blotting. RSK2 phosphorylated at Ser227, PDK1 phosphorylated at Ser241, anti-ERK1/2 phospho-44/42 were detected using specific antibodies for these proteins. Primary antibodies were visualized using secondary antibodies conjugated to either Alexa Fluor^®^ 680 (Thermo Fisher) or IRDye^®^ 800 (Li-Cor) for Odyssey imaging.

### Proximity ligation assay (PLA assay)

WT cells were grown in 10 mm confocal dish up to 70%–80% confluence (Corning), and serum starved for 24 h and then serum stimulated for 5 min followed by fixation, permeabilization, and blocking. Thereafter, Duolink PLA assay kit (Sigma Aldrich) protocol was followed for assessment of localization and co-association of RSK2 and MLCK. Two primary antibodies to RSK2 (anti-mouse) and MLCK (anti-rabbit) were used. PLA detects proteins within <40 nm.

### Western blots, antibodies and reagents

**FIGURE 1:**
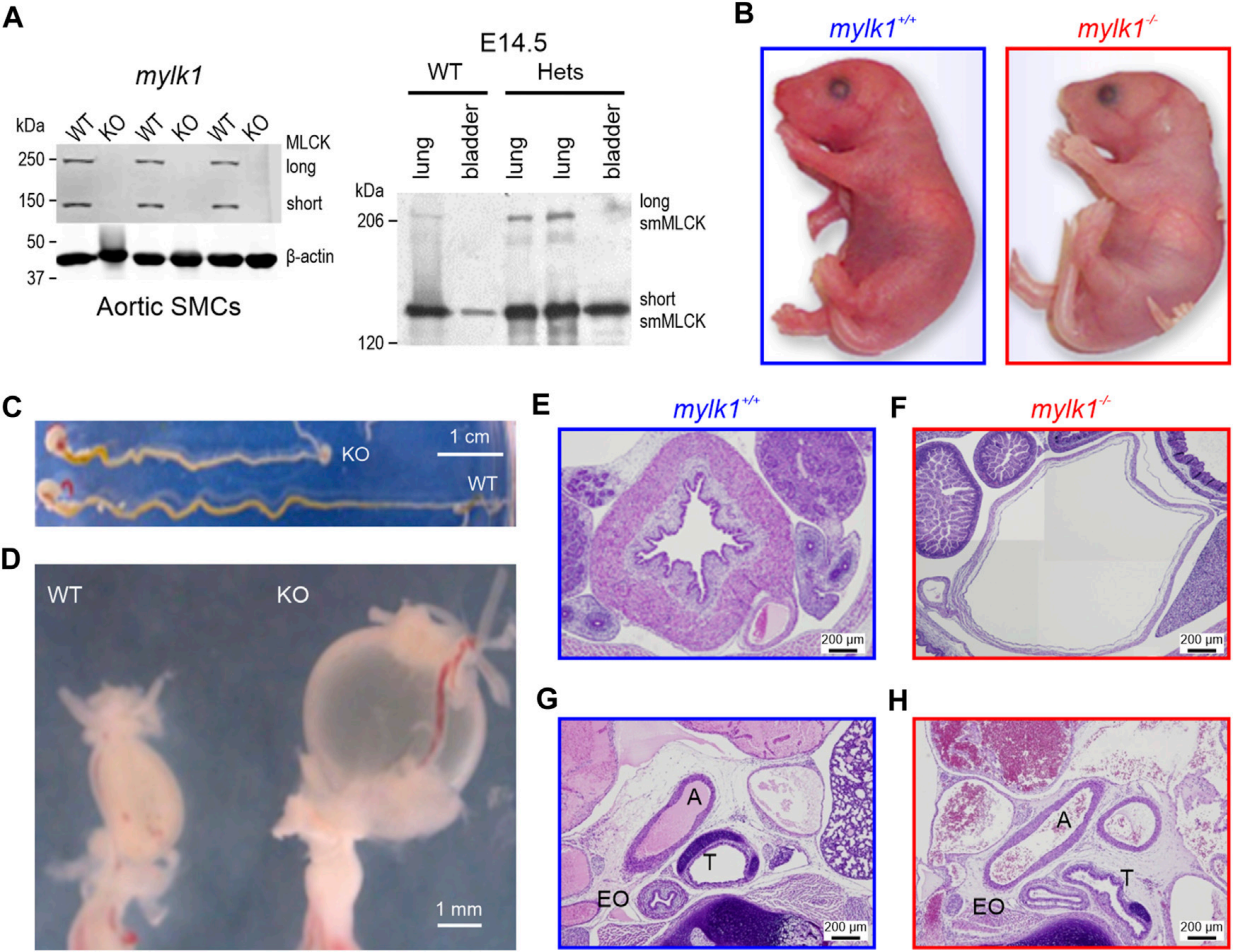
Long (220 kDa) and short (130 kDa) MLCK and telokin (17 kDa) protein expression and phenotypic differences in *mylk1* ^*−/−*^ and WT littermate mice. **(A)** Left panel: Western blots showing complete loss of MLCK isoforms 130 and 220 kDa in aortic SM cells cultured from E18.5 *mylk1* ^*−/−*^ mice. Loss of 17 kDa telokin is shown in Supplementary Figure S1B. Right panel: 220 kDa MLCK was not detected in E14.5 WT or *mylk* ^*+/−*^ bladder, with lung serving as a positive control. Proteins were subjected to SDS-PAGE followed by Western blotting with the anti–smMLCK (1:10000) antibodies and fluorescent Alexa Fluor 680 anti–mouse IgG secondary antibody (1:15000) and visualized by LICOR Imager. **(B)** Postnatal pups. *Mylk1* ^*−/−*^ died shortly after birth. *Mylk1* ^*−/−*^ pups are typically paler, the length of the gut, stomach to rectum is shorter **(C)** and the bladder is markedly enlarged **(D–F)**. Histology shows other dilated smooth muscle containing organs in transverse sections of E18.5 day embryos, stained with hematoxylin and eosin **(G,H)**. A, aorta; T, trachea; EO, esophagus. The image in **(F)** is composed of four images stitched together in order to match the magnification of the panels **(E,G,H)**.

Proteins were transferred to polyvinylidene difluoride (PVDF) or nitrocellulose membranes and blocked with Odyssey blocking buffer or 5%BSA, probed with primary followed by secondary antibodies in blocking buffer, and detected and quantified on the Odyssey system (LI-COR). Blots in Figure 1A, (right panel) only were detected by enhanced chemiluminescence (Amersham, Arlington Heights, Il) and quantitated using a Bio-Rad GS-670 imaging densitometer. The following antibodies were used: mouse monoclonal and rabbit polyclonal anti-actin (1:5000 WB; Sigma-Aldrich), MLCK (1:1000 WB; 1:250 PLA; 1:200 IP; Sigma-Aldrich and 1:500 IP; Abcam), goat polyclonal anti-RSK2 phospho-Ser227 and mouse monoclonal anti-RSK2 (1:500 WB; Santa Cruz, Biotechnology, Inc.). Mouse monoclonal anti-RLC_20_ (1:1000 WB; Sigma-Aldrich. Rabbit polyclonal anti-RLC_20_ phospho-Ser_19_ (1:200 WB), anti-ERK1/2 phospho-44/42 (1:500 WB), anti-PDK1-phospho-Ser241 (1:500 WB), anti-Pyk2 phospho-Tyr402 (1:500) from Cell Signaling. For triple westerns proteins were separated on 1.5 mm thick, 10% polyacryamide gel at 70 V for 3 h, transferred on 0.2 μm PVDF membrane at 100 V for 2 h in transfer buffer containing 25 mmol L^−1^ Tris-HCl, 192 mmol L^−1^ glycine, 1% SDS and 20% methanol. Membrane was blocked in 5% BSA in 0.01% TBST, cut at 75 kDa molecular mass marker and incubated overnight with p-Pyk2 Tyr402, p-PDK1 Ser241 antibodies at 4°C. Signal was enhanced using a triple Western blot protocol with biotin conjugated goat antirabbit IgG and Alexa Fluor 680 conjugated S-32358-streptavidin (1:100,000) (Johnson et al., 2009) and 31820-Goat anti-rabbit IgG (H + L) secondary antibody Biotin IgG (1:100,000) from Thermo Fisher Scientific were used.

### Statistical analysis

Data are expressed as mean ± SEM and analyzed by using either one-way ANOVA or two-way ANOVA followed by Bonferroni’s *post-hoc* test. The *p*-value of less than 0.05 was considered significant. Graphs were plotted using Graph Pad Prism version 9 software (San Diego, California, United States).

## Results

*Generation of MLCK/telokin KO mouse*: While generating the telokin-null mouse by homologous recombination (Khromov et al., 2006), we found that insertion of the full-length targeted construct into this locus, including the loxP flanked neomycin selection cassette, resulted in complete disruption of the *mylk1* gene expression in the absence of Cre recombination (Somlyo et al., 2004). The mice died shortly after birth. At E14.5 the litters (n = 125) showed close to the expected Mendelian inheritance ratio of 1.5^−/−^:4.8^+/−^:1^+/+^ with a normal average litter size of 8. Embryonic *mylk1* ^*−/−*^ aortic SM cells showed a complete absence of 220 and 130 kDa isoforms of MLCK (Figure 1A left panel) as evidenced by Western blotting. 17 kDa telokin is also absent in *mylk1* ^*−/−*^ aortic SM cells (Supplementary Figure S1B). In tissues the long form of MLCK is not expressed in either E18.5 embryonic WT or *mylk1* ^*+/−*^ bladder with lung serving as a positive control. The short 130 kDa isoform of MLCK, is present as expected. Telokin, a non-kinase SM-specific 17 kDa protein, identical to the C-terminus of MLCK is also absent in the *mylk1* ^*−/−*^. Telokin is phosphorylated by PKA/PKG (Wu et al., 1996; Wu et al., 1998; Khromov et al., 2006). When phosphorylated telokin binds to the phosphorylated myosin phosphatase facilitating its binding to phosphorylated myosin and promoting myosin dephosphorylation and relaxation (Khromov et al., 2012). The lack of telokin in the *mylk1* ^*−/−*^ embryos and SM cells would tend to increase RLC_20_ phosphorylation and basal force levels, yet SM containing tissues appeared dilated (Figure 1) and calyculinA forces were not different in WT and *mylk1* ^*−/−*^ SM. Furthermore, resting basal cyclic nucleotide-induced phosphorylation of telokin should be low (Renna et al., 2013). Telokin expression is also fivefold lower in vascular SM, used as a source of cultured embryonic SM cells, and for some contractility studies, compared to gut smooth muscle (Khromov et al., 2006).

### Phenotypic changes in *mylk1* ^*−/−*^ tissues

In general terms, the *mylk1* ^−/−^ embryos were physically indistinguishable from WT and heterozygous littermates (Figure 1B). Pups at birth, or embryos at E18.5 following dissection from the uterus, are pale in skin color as compared to both wildtype and heterozygous littermates. In addition, newborn KO animals tended to gasp for air within 2 h following delivery and prior to death. However, the lungs do inflate and there is closure of the ductus arteriosus following birth determined by visualization under a light microscope. Histological analysis showed dilation of bladders, aortae, trachea and esophagus (Figures 1D–H) consistent with a loss of SM tone in the absence of MLCK. Additionally, the length of the intestines from stomach to anus was shorter in the E18.5 *mylk1* ^−/−^ embryos compared to wild type (Figure 1C *p* < 0.0005). The bladders were always engorged, reflecting the loss of basal SM tone and bladder sphincters were patent (Figure 1D).

### *Mylk1* ^−/−^ smooth muscle retains contractile activity

**FIGURE 2:**
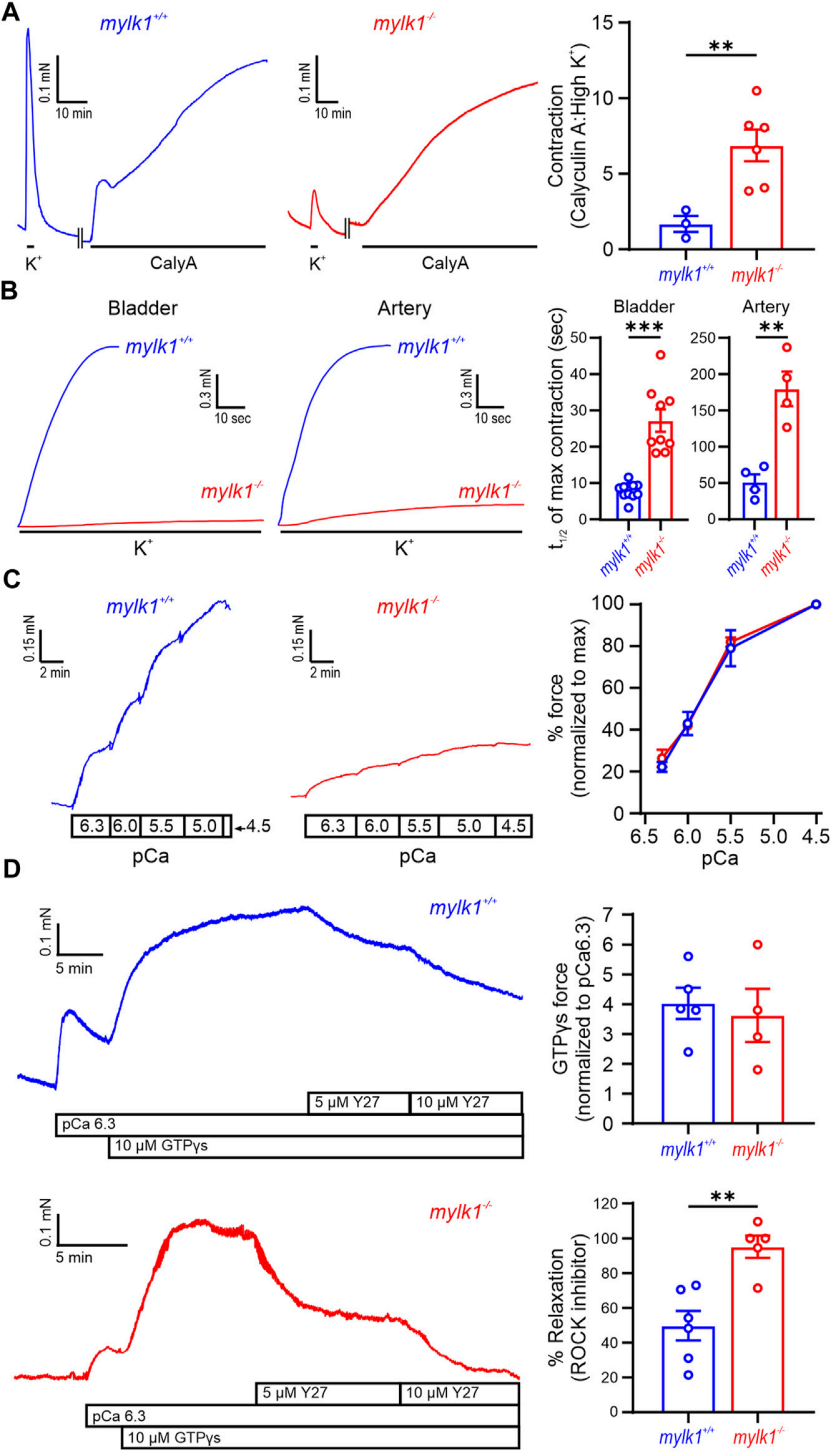
Assays demonstrating that *Mylk* ^*−/−*^ SM retained contractile activity: **(A)** Umbilical arteries from WT and *mylk1* ^*−/−*^ E18.5 embryos developed different magnitudes of force in response to high [K^+^]. Following permeabilization with α-toxin, phosphatase inhibitor calyculin A (100 nM) induced comparable magnitudes of maximal force in the *mylk1* ^*−/−*^ and the WT littermate vessels in pCa > 8 EGTA buffered solution. Ratios of calyculin A high [K^+^] in the graph demonstrate the reduced high [K^+^] compared to maximal force in the *mylk1* ^*−/−*^ compared to WT. **(B)** Different rates of contraction in response to high [K^+^] occur in WT compared to *mylk1* ^*−/−*^ in both bladder and umbilical arteries. Graph, the half-time of high [K^+^] contractions in *mylk1* ^*−/−*^SM strips was significantly greater than wildtype in both bladder (*p* < 0.0001) and arteries (*p* < 0.05). **(C)** *Mylk1* ^*−/−*^ SM contracts in responses to increasing [Ca^2+^]_i_, in α-toxin permeabilized bladder E18.5 *mylk1* ^*−/−*^ vs. WT SM strips. Left panel, pCa-tension responses. Graph, pCa-tension curve normalized to force at pCa 4.5 (100%). The magnitude of contractile force was less but the sensitivity to Ca^2+^ was not significantly different in *mylk1* ^*−/−*^ vs. WT bladder SM (*n* = 10 and 3 biological replicates respectively). **(D)** Activation of the RhoA/ROCK signaling pathway by addition of 10 μM GTPγS to α-toxin permeabilized WT and *mylk1* ^*−/−*^ bladder in pCa 6.3 solution. The ROCK inhibitor, Y27632, 5 and 10 μM relaxed the GTPγS-induced contractions. Graphs showed that the magnitude of GTPγS-induced contractions did not significantly differ between *mylk1* ^*−/−*^ and WT SM (*n* = 4 and 5 biological replicates respectively) but that the Y27632-induced relaxation of GTPγS-induced contractions was greater in the *mylk1* ^*−/−*^ SM compared to WT (*p* < 0.01, *n* = 5 and 6 biological replicates respectively).

The initial phasic component of the contractile response to high [K^+^] was approximately 5 times greater and significantly faster in the WT than the *mylk1* ^−/−^ embryonic bladder and umbilical arteries (Figures 2A, B). This suggests that upon depolorization, activation of MLCK makes a significantly larger contribution to the phasic component of high [K^+^] -induced contractions than the auxiliary non-MLCK kinase(s), which exhibit slower kinetics. However, the auxiliary kinase(s) in the absence of calcium is capable of inducing maximal forces equivalent to WT SM when exposed to the myosin phosphatase inhibitor, calyculin A, which allows for full RLC_20_ phosphorylation (Figure 2A). When SM was permeabilized with α-toxin, sensitivity to increasing intracellular [Ca^2+^] was not different between WT and *mylk1* ^−/−^ (Figure 2C), although the maximal force obtained at pCa 4.5 was significantly lower in the *mylk1* ^−/−^: (WT 9.5 ± 1.5 SEM cm vs. *mylk1* ^*−/−*^ 3.4 ± 0.5 cm, Supplementary Figure S2). The small GTPase RhoA, known to significantly contribute to contraction of SM through activation of ROCK and inhibition of MLCP (Somlyo and Somlyo, 2003), was also investigated to determine whether this signaling pathway is altered in m*ylk1* ^−/−^ mice. Addition of the non-hydrolyzable GTPγS—to generate active GTPγS*RhoA—to α-toxin permeabilized MLCK^−/−^ and WT SM, in the presence of pCa 6.3, contracted the muscles and the contraction was reversed by subsequent addition of the ROCK inhibitor Y27632 (Figure 2D). The greater Y27632-induced relaxation found in the *mylk1* ^−/−^ muscles compared to WT likely reflects the different ratios of MLCK:MLCP activity. Thus, MLCP reactivated by the ROCK inhibitor is dominant in the *mylk1* ^−/−^ muscles, resulting in a greater magnitude of relaxation.

### Myosin RLC_20_ phosphorylation occurs upon stimulation of *mylk1* ^−/−^ SM and inhibition of RSK2 suppresses this RLC_20_ phosphorylation

**FIGURE 3:**
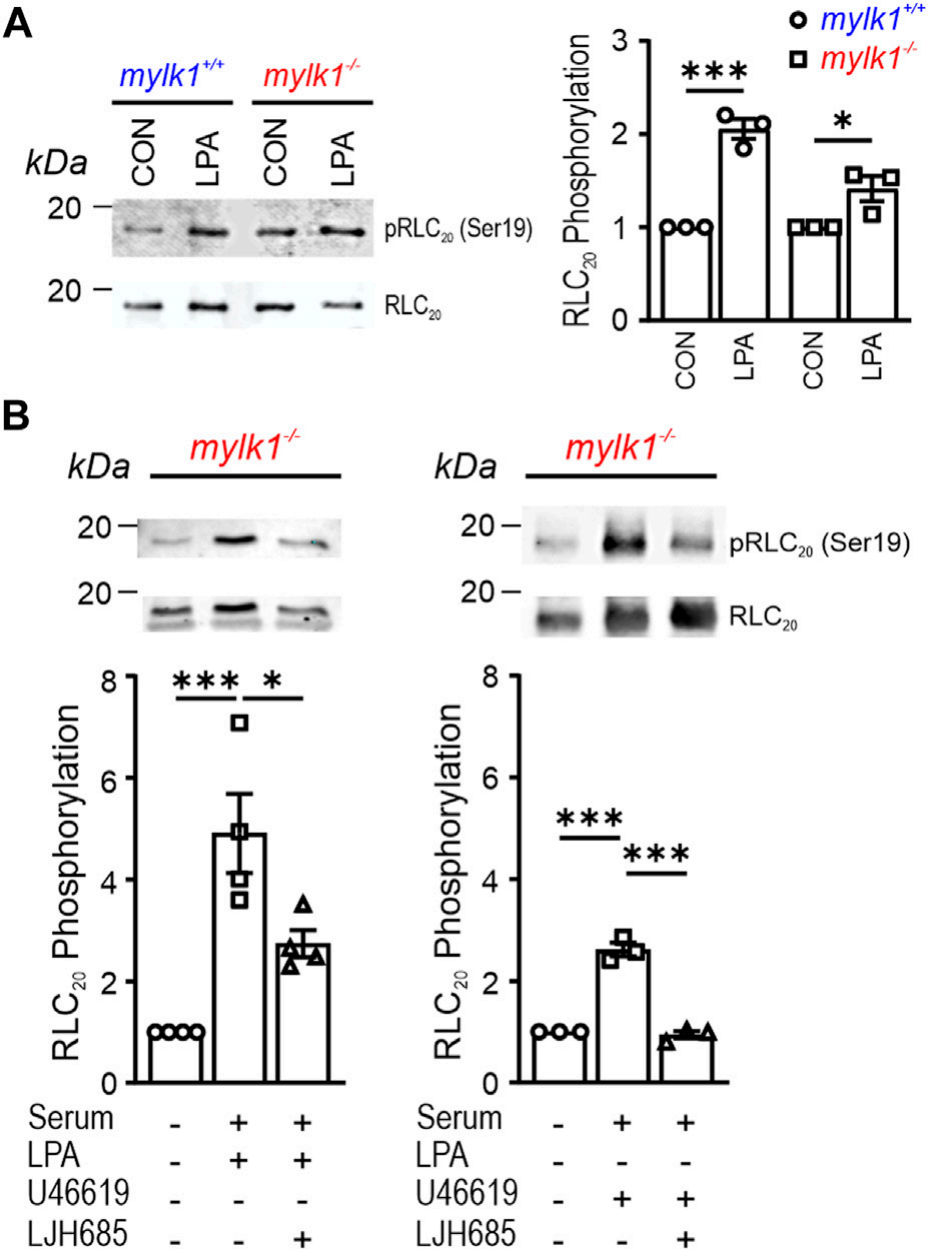
RSK2 kinase mediates myosin RLC_20_ phosphorylation in *mylk1* ^−/−^ SM cells. **(A)** LPA stimulation of cultured embryonic aortic *mylk1* ^*−/−*^ and *mylk1* ^*+/+*^ cells resulted in an increase in RLC_20_ phosphorylation (*n* = 3 biological replicates). **(B)** RSK inhibitor, LJH685, significantly inhibited RLC_20_ phosphorylation in *mylk1* ^*−/−*^ SM cells stimulated with LPA (*p* < 0.01, *n* = 4 biological replicates) or with thromboxane analogue, U47619 (*p* < 0.001, *n* = 3 biological replicates).

Based on the ability of *mylk1* ^−/−^ SM to contract, we next examined whether phosphorylation of myosin RLC_20_—a necessary step for activation of myosin crossbridge cycling—occurs upon contraction of *mylk1* ^−/−^ SM. LPA stimulation of cultured embryonic aortic *mylk1* ^*−/−*^ and *mylk1* ^*+/+*^ cells resulted in an increase in RLC_20_ phosphorylation in both the presence and absence of MLCK (Figure 3A). Stimulation of serum starved cultured aortic cells from *mylk1* ^−/−^ SM with serum plus LPA or the thromboxane analogue, U46619 significantly increased RLC_20_ phosphorylation, that was inhibited by RSK2 inhibitor, LJH685, a potent, ATP-competitive, and selective RSK2 inhibitor (Figure 3B). We also established that our cultured *mylk1* ^−/−^ cells were typical of SM cells as they expressed SM MHC (Suppl Data Figure 1), and actin. As RLC_20_ phosphorylation is a key event for initiating crossbridge cycling in SM, the increased RLC_20_ phosphorylation in the *mylk1* ^−/−^ SM demonstrates the presence of another kinase(s) capable of inducing RLC_20_ phosphorylation, accounting for the contractile capabilities of *mylk1* ^−/−^ SM. Inhibition by the RSK2 inhibitor further supports our hypothesis that RSK2 is the kinase largely responsible for contractile function in the *mylk1* ^−/−^ SM.

### Inhibition of RSK2 kinase suppresses carbachol-induced contractility of *mylk1* ^*−/*−^ bladder SM

**FIGURE 4:**
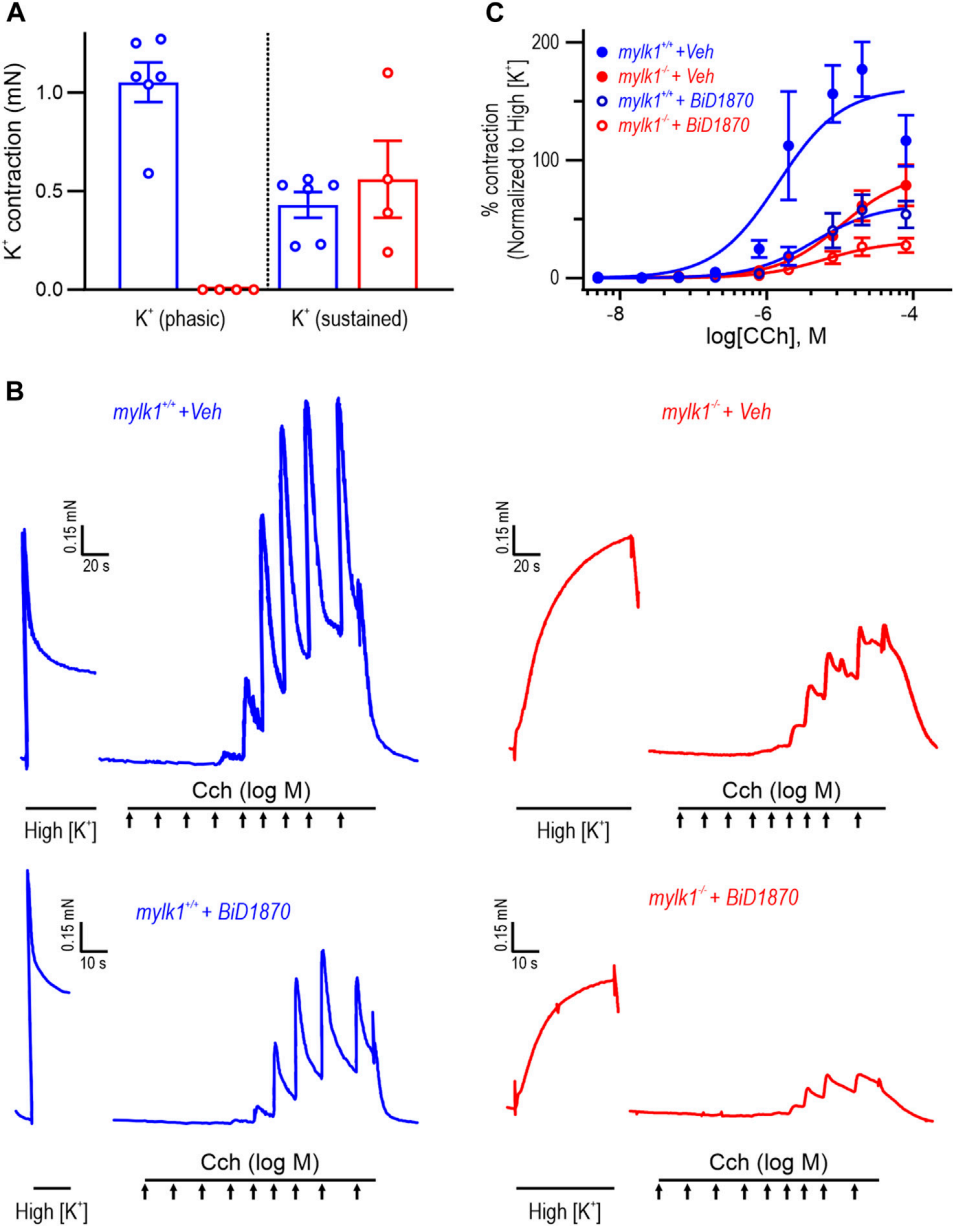
RSK inhibitor BiD1870 inhibited contractile responses to carbachol in *mylk1* ^−/−^ and WT bladder SM. **(A)** Summary of magnitude of high [K^+^]-induced phasic and tonic components of contraction in WT (*n* = 6) and *mylk1* ^−/−^ (*n* = 4) bladder SM. The high [K^+^] phasic component was absent or small in the *mylk1* ^−/−^ while the tonic components present in embryonic bladder did not differ significantly in magnitude *p* = 0.5. **(B)** The contractile response to high [K^+^] was recorded and following return to high [Na^+^] Hepes buffered Krebs solution the muscles were treated with increasing concentrations of carbachol (Cch) in the presence or absence of the RSK inhibitor, BiD 1870 (1 μM) or equivalent volumes of diluent, DMSO. **(C)** Contractile responses to increasing concentrations of Cch were normalized to the tonic high [K^+^] contractions for each muscle and analyzed by 2-way Anova. WT DMSO vs. WT BiD 1870: *p* < 0.0001, *mylk1* ^−/−^ DMSO vs. *mylk1* ^−/−^ BiD 1870: *p* < 0.003, *n* = 3 WT, *n* = 2 *mylk1* ^−/−^. Arrows = incremental Cch concentrations from 5 × 10^−9^ to 5 × 10^−5^.

High [K^+^] induced contractions induces an initial phasic component followed by a tonic component in embryonic bladder. This tonic component was absent or very small in umbilical arteries as shown in Figure 2B. In *mylk1* ^−/−^ bladder SM the phasic component was absent or very small while the tonic components were present and of similar magnitude in the WT and *mylk1* ^−/−^ bladder SM (Figures 4A, C). The tonic component was used to normalize the responses to carbachol (Figure 4B). Contractile responses to increasing concentrations of carbachol were significantly inhibited in the presence of the RSK2 inhibitor BI-D1870 compared to the DMSO diluent in both *mylk1* ^−/−^ and WT bladder SM, *p* < 0.0001 and *p* < 0.003, respectively (Figure 4B). Force was not completely abolished by BI-D1870 in the *mylk1* ^−/−^ tissues.

### RSK2 colocalizes and co-immunoprecipitates with MLCK and actin upon stimulation in aortic SM cells

**FIGURE 5:**
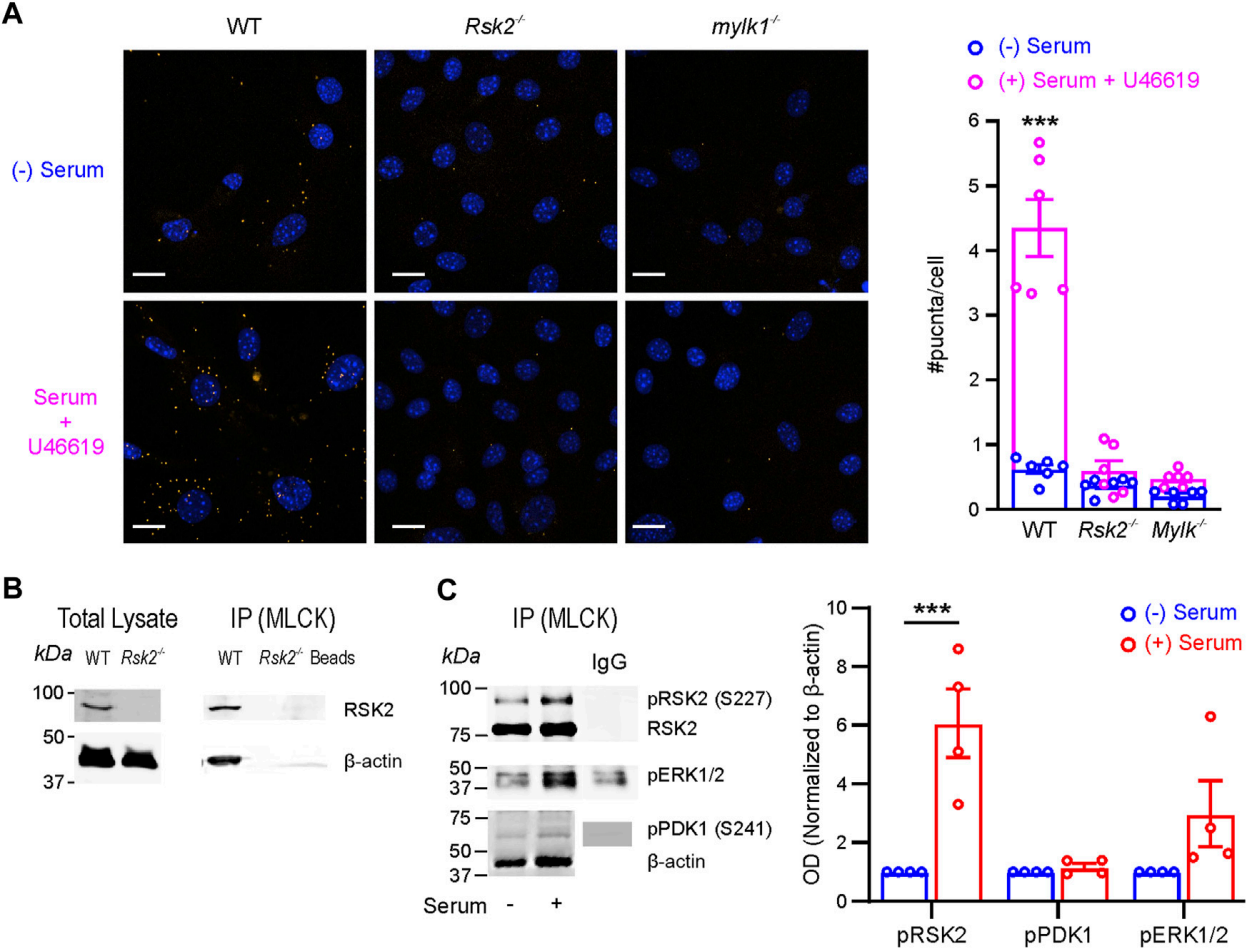
RSK2 and upstream activators, ERK1/2 and PDK1 associate with MLCK and actin in SM cells: **(A)** RSK2 and MLCK colocalized in the proximity ligation assay (PLA) in WT, but not RSK2^−/−^ and *mylk1* ^−/−^ SM cells, in both serum starved or in the presence of serum plus the thromboxane analogue, U46619. Cells were labeled with rabbit monoclonal anti-MLCK antibody (1:200) and mouse monoclonal anti-RSK2 antibody (1:200), followed by secondary antibodies coupled to oligonucleotides (PLA probes). Following amplification of PLA probes in close proximity, complementary oligos coupled to fluorochromes hybridized to the amplicons and gave rise to discrete fluorescent puncta. Blue nuclei are stained with DAPI. The number of puncta/cell was significantly greater in the serum plus U46619 stimulated WT cells compared to non-stimulated (*p* < 0.001 *n* = 3 biological replicates) and to a lesser extent in non-stimulated WT cells compared to non-stimulated *mylk1* ^−/−^ SM cells (<0.05 *n* = 3 biological replicates). RSK^−/−^ and *mylk1* ^−/−^ cells served as negative controls and had few puncta with or without stimulation. **(B)** MLCK immunoprecipitation (IP). RSK2 and actin were immunoprecipitated by MLCK in WT but not RSK2^−/−^ SM cells or beads alone. Neither RSK2, MLCK or actin were immunoprecipitated by IgG alone (Supplementary Figure S1C). **(C)** Representative Western blot and graph showing MLCK IP from mouse WT aortic SM cells in the absence or presence of serum. RSK2, phosphorylated RSK2^S227^, phosphorylated ERK1/2 and phosphorylated PDK1Ser^241^ as well as actin were immunoprecipitated by MLCK in both the presence and absence of serum (*n* = 4 biological replicates). Serum conditions significantly increased the amount of phosphorylated RSK2^S227^ immunoprecipitated by MLCK *p* < 0.001, while there was an increased trend with phosphorylated ERK1/2. Phosphorylated proteins were normalized to actin and 0 serum conditions taken as 1 for each phosphorylated protein in the graph.

The proximity ligation assay (PLA) indicates that RSK2 colocalized within less than 40 nm distance from MLCK and that this association increased 4-fold upon stimulation with serum plus the thromboxane analogue, U46619 (Figure 5A). RSK2^−/−^ and MLCK^−/−^ cells served as negative controls. This association of RSK2 and MLCK was confirmed in immunoprecipitation assays (Figure 5B), where MLCK antibody pulled down RSK2, in WT but not RSK2^*−/−*^ SM cells. Actin was also pulled down. Beads alone served as a control and were negative as was IgG (Supplementary Figure S1C). MLCK immunoprecipitation also pulled down phosphorylated RSK2Ser^227^, RSK2, phosphorylated ERK1/2, phosphorylated PDK1Ser^241^, and actin (Figure 5C). Serum stimulation significantly increased the association of MLCK with phosphorylated RSK2Ser^227^ with a tendency for an increase in phosphorylated ERK1/2, an upstream activator of RSK2.

### Ang II activates Ca^2+^-dependent tyrosine kinase, Pyk2 and the Pyk2 inhibitor (PF-4618433) inhibits activation of both Pyk2 and its downstream target PDK1

**FIGURE 6:**
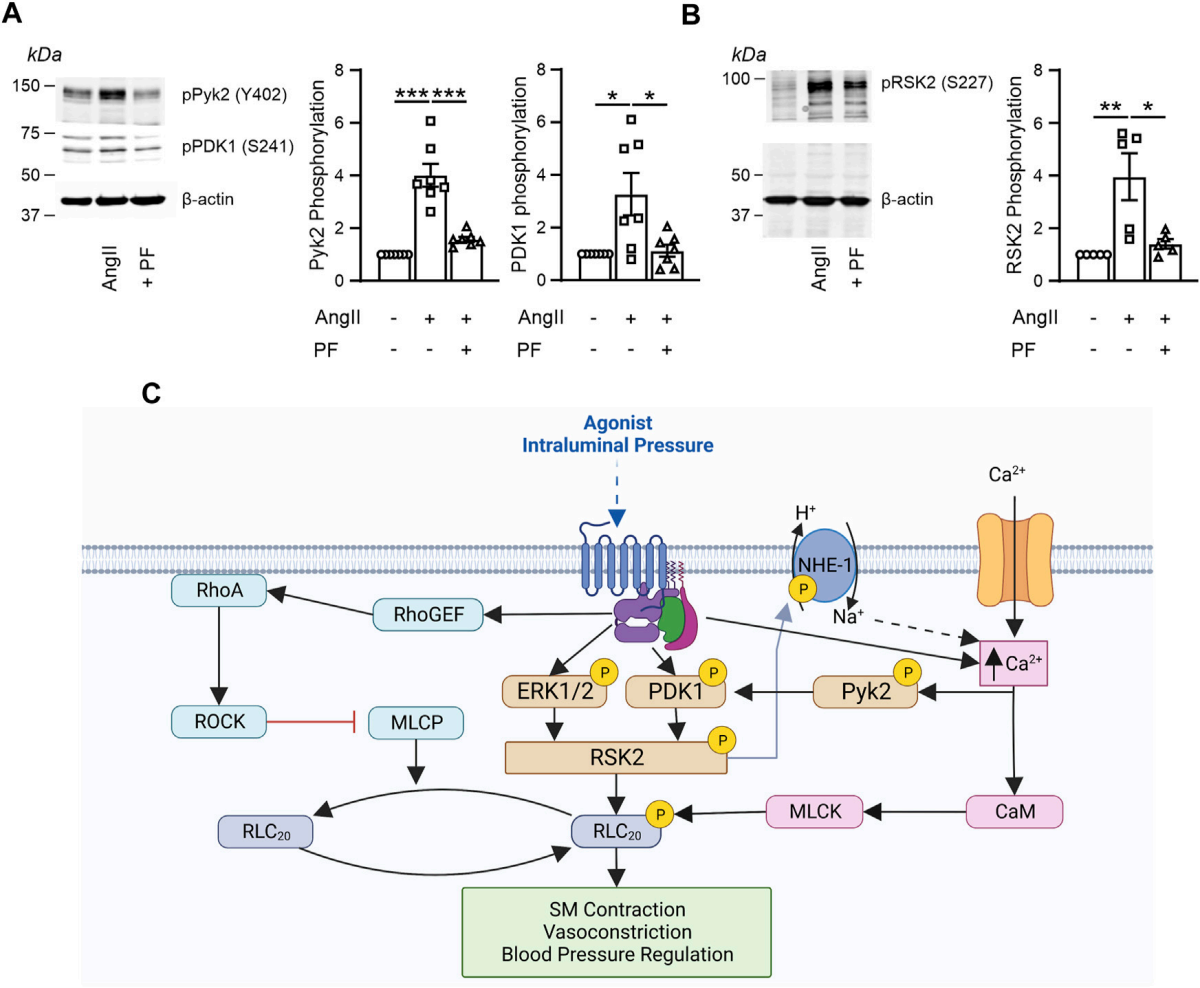
AngII stimulation increased phosphorylation of the Ca^2+^ dependent tyrosine kinase, Pyk2 and of its downstream targets PDK1 and RSK2 which was suppressed by Pyk2 inhibitor, PF-4618433. **(A)** Representative Western blot and graphs from cultured E18.5 aortic SM cells showing significant AngII-induced phosphorylation of both Pyk2 (*n* = 8 biological replicates, *p* < 0.001) and PDK1 (*n* = 7 biological replicates, *p* < 0.01). The Pyk2 inhibitor, (PF-4618433), inhibited activation of both Pyk2 and its downstream target, PDK1 (*n* = 8, *p* < 0.001 and *n* = 7, *p* < 0.01 respectively). **(B)** AngII increased RSK2 phosphorylation (*n* = 5, *p* < 0.05) that was significantly inhibited by PF-4618433 (n = 5, *p* < 0.01). Proteins normalized to actin. **(C)** Three signaling pathways regulating SM RLC_20_ phosphorylation, contractility, basal tone and regulation of blood pressure. Agonists, neurotransmitters or increases in arterial intraluminal pressure activate; 1) the canonical Ca^2+^ activated MLCK pathway. 2) the RhoA/ROCK pathway that phosphorylates and inhibits MLCP resulting in an increase in RLC_20_ phosphorylation. 3) the new RSK2 pathway that leads to activation of NHE-1 resulting in cytosolic alkalinization and increase in Ca^2+^ events. This increased Ca^2+^ in addition to Ca^2+^influx and release from stores, feeds back to further activated the MLCK pathway. Activated RSK2 can also directly phosphorylate RLC_20_. Different stimuli may preferentially select for a given signaling pathway. Created by BioRender.com.

Agonist stimulation of aortic SM cells significantly increased Pyk2 phosphorylation, (Figure 6A), that was significantly inhibited by pretreatment with PF-4618433. Phosphorylation of the RSK2 downstream target, PDK1, known to phosphorylate S227 and activate RSK2, was also significantly inhibited by the Pyk2 inhibitor, as was phosphorylation of RSK2 S227 (Figure 6B).

## Discussion

Pathophysiology of vascular smooth muscle (VSM) constitutes a critical element of the etiology of hypertension. Specifically, increased sustained vascular contraction, i.e., higher tone, leads to increased vascular resistance due to reduced vascular diameter and arterial remodeling (Renna et al., 2013). Hypertension is a major risk factor for heart failure, myocardial infarction, stroke, vascular dementia, and other diseases, but the current pharmacotherapiesrelying primarily on diuretics, β-blockers, Ca^2+^-channel blockers and renin-angiotensin pathway inhibitors—have limited success rates, leaving behind a significant number of patients refractory to treatments. It is not surprising that molecular mechanisms governing VSM contraction are therefore actively studied in search of alternative drug targets. Most relevant cellular signaling pathways are regulated by protein phosphorylation, catalyzed by specific kinases. The latter are attractive drug targets, and many kinase drugs have been approved in the last two decades for clinical use, especially to treat cancers (Cohen, 2002; Attwood et al., 2021). And the field of design of kinase inhibitors for use in the clinic is highly advanced, with 68 approved by FDA as of 2022 (Roskoski, 2022). Therefore, kinases involved in VSM regulation might potentially offer new avenues for the treatment of hypertension.

As described in the *Introduction*, recent studies showed that RLC_20_ is a substrate for phosphorylation by protein kinases other than MLCK1. The fact that complete global deletion of the *mylk1* gene does not prevent formation of embryos—even though they die after birth (Somlyo et al., 2004)—provides compelling evidence that auxiliary kinases activating SM myosin are physiologically important in SM, and capable for compensating for lack of MLCK1. However, auxiliary kinases could not substitute for all MLCK functions as SM containing tissues such as aorta, bladder, trachea and esophagus from *mylk1* ^*−/−*^ mice were markedly dilated. Dilated irregular subepicardial coronary vessels were observed from *mylk1* ^*−/−*^ mice. Here, we expand the characterization of the *mylk1* ^−/−^ global knockout, and demonstrate that SM tissues including bladder, fundus and blood vessels, as well as cultured SM cells, retain the ability to contract and migrate through the normal pathway, as evidenced by increased RLC_20_ phosphorylation in response to agonists (Figure 3). Importantly, addition of the MLCP inhibitor, calyculin A, to *mylk1* ^−/−^ arteries and bladder in the absence of Ca^2+^ induces contractions of similar magnitude to those seen in normal arteries of similar size (Figure 2A). Therefore, under these conditions, the auxiliary RLC_20_ kinase is able to induce the full force potential of SM, and indicates that the contractile apparatus is intact. Based on the calyculinA contractions, this kinase appears to be Ca^2+^-independent and operating in *mylk1* ^−/−^ tissues, in the presence of intact RhoA/ROCK pathway (Scheme, Figure 6C), which is also fully functional because embryonic SM tissues contract to GTPγS stimulation at constant [Ca^2+^] and inhibition of ROCK relaxes the GTPγS contractile responses (Figure 2D).

The percent relaxation due to the increased phosphatase activity, upon inhibition of ROCK, was significantly greater in the *mylk1* ^−/−^ SM as the magnitude of contractile force depends on the ratio of kinase to phosphatase activity. RSK2 kinase activity is less and slower than MLCK, altering this ratio and allowing a preponderance of ROCK activated phosphatase activity. Likewise, the smaller and slower initial phasic high [K^+^] contraction in the *mylk1* ^−/−^ compared to the *mylk1* ^+/+^ arteries reflects the depolarization induced rapid Ca^2+^ transient that activates Ca^2+^/CaM/MLCK present in the *mylk1* ^+/+^ but lacking in the *mylk1* ^−/−^ arteries (Figures 2A, B).

The challenge now is to identify the most relevant protein kinase in VSM that might constitute a good target for therapy. We recently presented strong evidence supporting the notion that this role is played by the p90 ribosomal S6 kinase (RSK2), coded by the *RPS6KA3* gene, which has been hitherto recognized primarily for its role in brain development [*RPS6KA3* mutations cause the Coffin-Lowry developmental syndrome (Hanauer and Young, 2002; Jacquot et al., 2002)] and cancer progression [it is a recognized target for cancer therapy, particularly the triple-negative breast cancer (Huynh et al., 2020)]. Specifically, previously we used SM tissues from global *RPS6KA3* ^*−/−*^ mice, which are viable, to demonstrate the impact of deletion of RSK2 from SM (Artamonov et al., 2018). In the present, complementary study, we further explored the role of RSK2 in the SM tissues from *mylk1* ^−/−^ embryos.

Arguably the most direct and compelling confirmation of our hypothesis in the present study is the fact that specific RSK2 inhibitors, Bi-D1870 and LJH685, inhibited carbachol-induced contractility in bladder of the *mylk1* ^−/−^ embryos (Figure 4), as well as RLC_20_ phosphorylation on Ser (Carlson et al., 2018) in *mylk1* ^−/−^ SM cells stimulated with either LPA or U46619 (Figure 3). Therefore, in the absence of MLCK, contractility appears to be regulated primarily by the activities of RSK2 and the RhoA/Rock signaling pathways (Scheme, Figure 6C). The low, residual contractility and RLC_20_ phosphorylation still observed in the presence of the RSK2 inhibitors may be due to incomplete RSK2 inhibition or to other kinases such as RSK1, integrin-linked kinase (Deng et al., 2001; Wilson et al., 2005), PIM/ZIPK (Carlson et al., 2018), or IKK2 (Ying et al., 2013). Nevertheless, our results support the notion that RSK2 is the long sought primary auxiliary contributor to SM contractility, independent of MLCK activity.

In normal SM cells, stimulation activates both MLCK and RSK2. In this case, as we have shown previously (Artamonov et al., 2018). RSK2 can both directly phosphorylate RLC_20_ and also activate the Na^+^/H^+^ exchanger NHE-1 resulting in an alkalinization of the cytosol, an increase in [Ca^2+^] by a yet to be identified mechanism, and amplification of MLCK activity (Scheme, Figure 6C). When these pathways dominate over MLCP activity, the net result is constriction, as in the case of myogenic vasoconstriction induced by agonist or intraluminal pressure, or in maintenance of basal tone in the resting state. It is however, of importance to note that not all three pathways are activated equally by different stimuli, or have the same time course (Artamonov et al., 2013b). For example, thromboxane analogue U46619 is a specific potent activator of the RhoA signaling. On the other hand, the onset of depolarization with high [K^+^] is dominated by Ca^2+^/CaM/MLCK signaling as seen in Figure 2A when comparing initial high [K^+^] responses in the arteries from the normal and *mylk1* ^−/−^ mice. RSK2 signaling, in turn, contributes ∼25% of the myogenic vasoconstriction response in resistance arteries, and contributes to resting blood pressure control (Artamonov et al., 2018). These multiple pathways provide both redundancy but also specialization depending on the stimuli, resulting in phasic or tonic type contractions.

Although RSK2 activity appears to be independent of MLCK, in normal SM cells MLCK immunoprecipitates with RSK2 and actin and this association increased with stimulation. The co-localization was confirmed in PLA assays, showing they are <40 nm apart. MLCK has an actin-binding site in its N-terminus with its C-terminal kinase domain poised to reach across to interact with, and phosphorylate myosin heads to initiate myosin cross bridge cycling (Lin et al., 1999). We have previously reported that a fraction of RSK2 immunoprecipitates with actin filaments (Artamonov et al., 2018), while a soluble fraction is free to phosphorylate other substrates, such as the membrane associated Na^+^/H^+^ exchanger NHE-1. Interestingly, the two upstream activators of RSK2—PDK1 and ERK1/2— were also brought down with the immunoprecipitated complex of RSK2/MLCK and actin, suggesting a signaling hub on the actin filament poised to phosphorylate and activate neighboring myosin filaments. Few puncta were observed in the (−) serum PLA assay unlike the (−) serum immunoprecipitation assay where MLCK pulls down actin plus RSK2, ERK1/2 and PDK1. (+) serum increased the immunoprecipitation of RSK2 and phospho-RSK2. The immunoprecipitation assay requires homogenization of the sample which may have partially activated the associations unlike the PLA assay carried out on intact cells. In this case, RSK2, possibly complexed to ERK1/2 and PDK1 may be bound to actin and not directly to MLCK, so not detected in the unstimulated SM cells in the PLA assay. It remains to be determined, if the proximity of RSK2 to MLCK alters its activity and *vice versa*.

An intriguing question also remains as to how low (∼1 μM) concentrations of kinases tethered to actin filaments can phosphorylate ∼100 μM myosin heads. One reported possibility is that MLCK diffuses along actin filaments and enhances the rate of phosphorylation of SM myosin, as described by others (Hong et al., 2015). In that study, single molecules of fluorescent quantum dot labeled MLCK moved along actin filaments and preferentially located to areas in which myosin was not yet phosphorylated. The motion occurred only if the acto-myosin and MLCK-myosin interactions were weak. If the RSK2 associated with the MLCK/actin complex also moves along actin, this could similarly phosphorylate myosin heads provided that the RSK2 catalytic N-terminal domain can physically reach the myosin heads. Otherwise, the activated MLCK-associated RSK2 may regulate the activity of its MLCK partner.

One of our major concerns was that the results concerning [Ca^2+^] dependence of the auxiliary pathway were apparently contradictory. While the RSK2-mediated contractile force developed in *mylk1* ^−/−^ SM in response to calyculin A was independent of Ca^2+^ (pCa > 8), the permeabilized *mylk1* ^−/−^ SM contracted in response to increased [Ca^2+^] (Figure 2C), with maximal force at pCa4.5 being ∼3 fold lower in the *mylk1* ^−/−^ bladder. As the magnitude of contraction and RLC_20_ phosphorylation reflects the ratio of kinase:phosphatase activity and assuming phosphatase activity is constant, the MLCK activity present in the WT accounts for its greater force at pCa 4.5. We previously reported that the pCa-force relationship in α-toxin permeabilized rabbit arteries was right-shifted and the maximal [Ca^2+^] response was inhibited by as much as ∼ 60% by either the RSK2 inhibitor BI-D1870 or the PDK1 inhibitor GSK2334470. These data suggest Ca^2+^-dependence of RSK2 and PDK1. AngII stimulation of SM cells from human resistance arteries has been shown to increase intracellular [Ca^2+^] and to induce a cytosolic alkalinization, which leads to increased activities of ERK(1/2) and several tyrosine kinases (He et al., 1998). These changes in [Ca^2+^] and alkalinization are consistent with AngII activation of RSK2 signaling (Artamonov et al., 2018) and the tyrosine kinase activity suggest a role for another kinase. The non-receptor proline-rich tyrosine kinase Pyk2 is Ca^2+^-dependent and phosphorylates PDK1 in AngII stimulated vascular SM (Taniyama et al., 2003). Pyk2 has also been shown to play a role in contraction of arterial SM (Mita et al., 2013; Mills et al., 2015). Knockdown of PDK or Pyk2 inhibits LPA-induced phosphorylation of RSK2 and NHE3 activity in immortalized human colorectal adenocarcinoma cells (No et al., 2015). Similarily, in our studies, inhibition of Pyk2 inhibits AngII phosphorylation of Pyk2, PDK1 and RSK2 (Figure 6A). Activation of the RSK2 pathway, resulting in activation of NHE-1 and an increase in Ca^2+^ events (Taniyama et al., 2003), may also contribute to Pyk2 activation. Whether this increase in Ca^2+^ and/or the Ca^2+^ influx and release from stores provides the source for activation of Pyk2 remains to be determined. Using a highly selective inhibitor of Pyk2, PF4618433 (Han et al., 2009), we have found that AngII phosphorylates Pyk2 in aortic SM cells. PF4618433 inhibits both phosphorylation of Pyk2 and its downstream target PDK1 as well as its downstream target RSK2 phosphorylation. Altogether, these findings are consistent with Pyk2 being upstream of PDK1 and RSK2 and accounting for the observed Ca^2+^-dependent component of RSK2 activity (scheme, Figure 6C). Based on the magnitude of this Ca^2+^-dependent component in the absence of MLCK (Figure 2C), it accounts for approximately 25% of the maximal contractile force in the WT. Maximal force in response to CalyculinA inhibition of MLCP was equivalent in WT and *mylk1* ^*−/−*^ smooth muscles (Figure 2A).The Ca^2+^-independent component in the *mylk1* ^*−/−*^ *SM* reflects the RSK2 direct phosphorylation of myosin RLC_20_ and RSK2 activation of NHE-1 leading to increased Ca^2+^ events.

The limited tissue size, fragility and availability from the E18.5 embryos did not allow us to make a rigorous comparison of the impact of deletion of MLCK on the contractile properties of different types of SM. The histology showed that bladder, bronchi, trachea and blood vessels were dilated in the *mylk1* ^*−/−*^ embyros compared to WT littermates. We were able to compare contractile responses to high K^+^ treatment in bladder and artery (Figure 2B). In both cases the t_1/2_ was significantly slower in the *mylk1* ^*−/−*^ compared to WT SM but the bladder was faster than the artery, in both the presence or absence of MLCK. The tonic component of the high K^+^ stimuli predominated in the bladder (Figure 4A). All SM tissues and cells lacking *mylk1* ^*−/−*^ displayed evidence for an auxiliary kinase(s) other than MLCK that supports contractility, RLC_20_ phosphorylation and RSK2 signaling pathways. While determining the heterogeneity of different embryonic SM tissues responses was not feasible, all experiments supported our hypothesis that RSK2 kinase plays a significant role in SM contractility. The magnitude in different SMs remains to be determined. It is∼25% of maximal force in mouse resistance mesesenteric arteries (Artamonov et al., 2018).

In conclusion, we present compelling evidence that RSK2 largely accounts for the contractile and phosphorylated RLC_20_ myosin observed in response to stimuli in SM from the *mylk1* ^*−/−*^ embryos. These embryos have also allowed for the determination of the contributions of the RSK2 signaling to SM contractility in the complete absence of the canonical Ca^2+^/CaM/MLCK signaling. These findings support our previous studies showing the physiological importance of RSK2 signaling in the regulation of SM contractility and resting blood pressure. RSK2 contributes ∼25% of maximal force generated in response to increased intraluminal pressure in normal resistance arteries that regulate blood pressure and basal tone (Artamonov et al., 2018). Altogether, we propose that there are three major signaling pathways, driven by MLCK, RSK2 and RhoA/ROCK that regulate RLC_20_ phosphorylation and force in smooth muscle (Scheme, Figure 6C). The magnitude of their contributions depends on the stimuli and the smooth muscle tissue.

## Data Availability

The raw data supporting the conclusion of this article will be made available by the authors, without undue reservation.

## Supplementary material

SUPPLEMENTARY FIGURE S1
**(A)** SM myosin heavy chain, an established marker for SM cells and actin are present in cultured aortic SM from E18.5 *mylk* ^−/−^ and ^+/+^ aortae: Proteins were subjected to SDS-PAGE followed by western blotting with anti-SM myosin heavy chain (1:800), a gift from Dr. David Hartshorne, Univ. of Arizona) and β-actin (1:10,000). After a brief wash with diH2O, total protein was visualized with Revert^TM^ 700 Total Protein Stain (LI-COR) and imaged with a LI-COR Odyssey scanner. The total protein stain was removed using multiple washes with the Revert^TM^ 700 destaining solution (LI-COR). The total protein signal was quantified using Image Studio (LI-COR). The total protein stain was used as a loading control. **(B)** Expression of 130 kDa MLCK and 17 kDa telokin in aortic SM cells from *mylk1* ^−*/*−^ and WT littermate mice: 130 and 17 kDa products of *mylk1* were absent in *mylk1* ^−*/*−^ *aortic SM cells.* Proteins were subjected to SDS-PAGE followed by western blotting with the anti–smMLCK (1:10000) and anti–telokin (1:1000) antibodies and fluorescent Alexa Fluor 680 anti—mouse IgG secondary antibody (1:15000) and visualized by LI-COROdyssey Scanner. An unidentified non-specific band (NSB) served as a loading control. **(C)**: Control experiments for the MLCK immunoprecipitation experiments. IgG alone did not pull down MLCK or RSK2. A β-actin band was detected in the upper panel but the intensity was very faint compared to the actin pulled down in Figures 5B, C. MM, molecular weight marker.

SUPPLEMENTARY FIGURE S2 The magnitude of contractions in α-toxin permeabilized bladder E18.5 *mylk1* ^−*/*−^ vs. WT SM strips was measured in response to increasing concentrations of Ca^2+^ from pCa 6.3 to 4.5. Maximal contractions are significantly smaller in the *mylk1* ^−*/*−^ (pCa6.3: 1.0 ± 0.2 cm, pCa6.0: 1.4 ± 0.2 cm, pCa5.5: 2.8 ± 0.4 cm, pCa4.5: 3.4 ± 0.5 cm; n = 8–10 biological replicates) compared to WT littermate bladders (pCa6.3: 2.2 ± 0.5 cm, pCa6.0: 4.2 ± 1.1 cm, pCa5.5: 7.6 ± 1.8 cm, pCa4.5: 9.5 ± 1.5 cm; *n* = 3 biological replicates; **p* < 0.05; ****p* < 0.001).
