# Supplementary figures and images for: p90RSK2, a new MLCK mediates contractility in myosin light chain kinase null smooth muscle
Source: Front Physiol. 2023 Sep 13;14:1228488. doi: 10.3389/fphys.2023.1228488 (PMC10533999; doi:10.3389/fphys.2023.1228488)

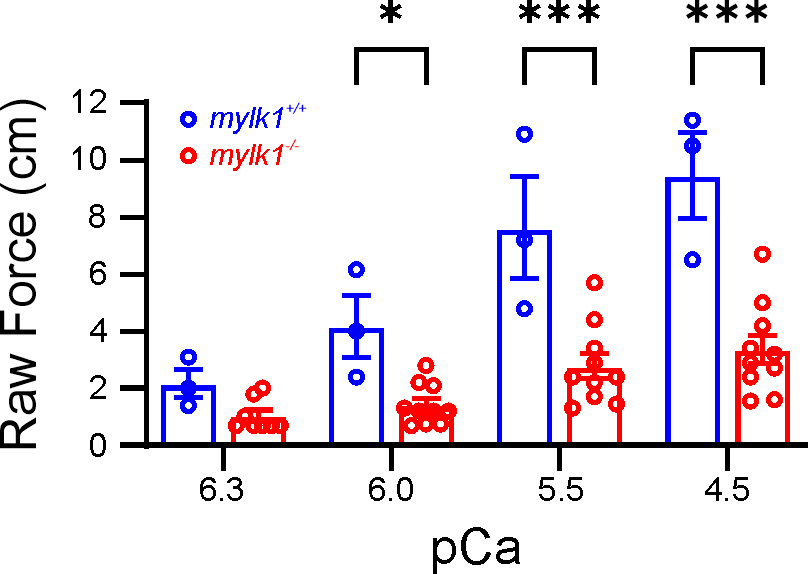

Supplement: Supplementary file 1 [file Image2.tif]

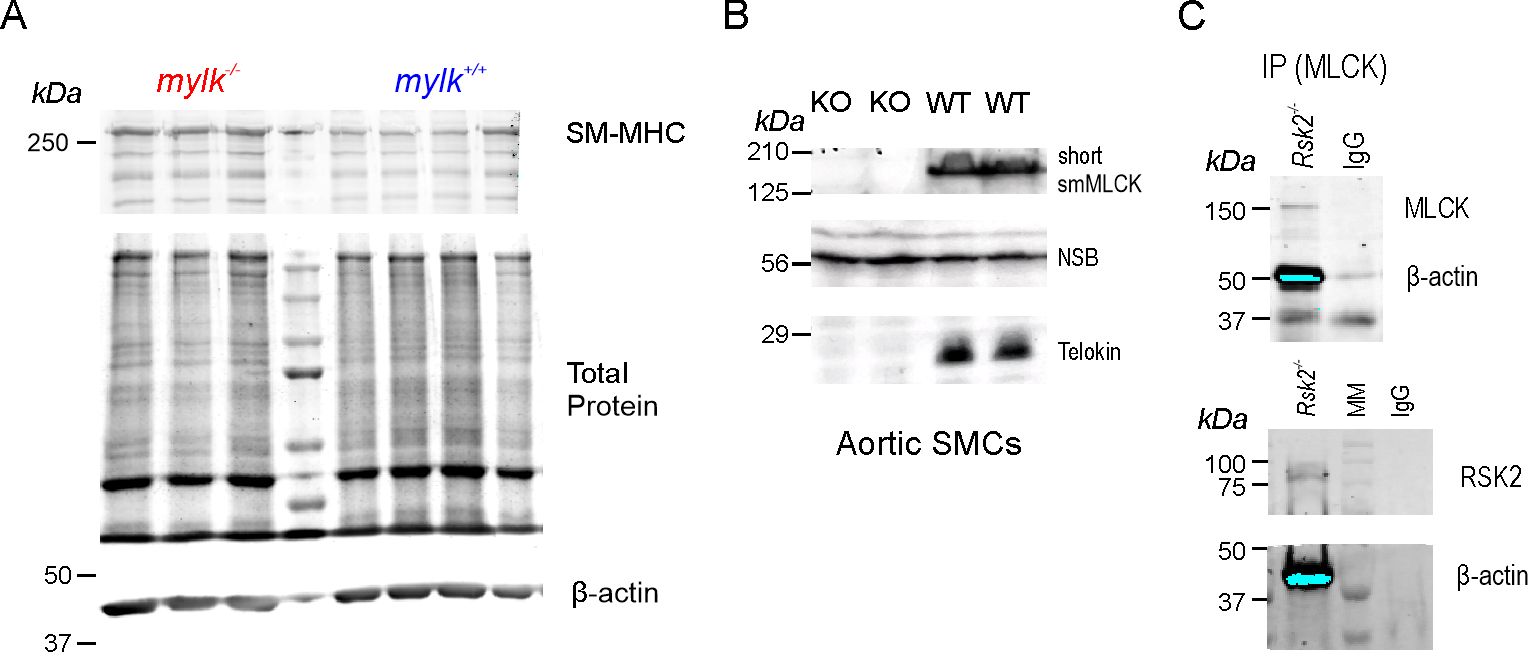

Supplement: Supplementary file 2 [file Image1.tif]
